# Elevated preoperative red cell distribution width and incident anemia after metabolic and bariatric surgery: a cohort study

**DOI:** 10.3389/fnut.2026.1863689

**Published:** 2026-07-13

**Authors:** Kuo-Chuan Hung, Ting-Sian Yu, I-Wen Chen

**Affiliations:** 1 Department of Anesthesiology, Chi Mei Medical Center, Tainan City, Taiwan; 2 Department of Anesthesiology, E-Da Hospital, I-Shou University, Kaohsiung City, Taiwan; 3 Department of Anesthesiology, Chi Mei Hospital, Liouying, Tainan City, Taiwan

**Keywords:** iron deficiency anemia, metabolic and bariatric surgery, postoperative anemia, red cell distribution width, risk stratification

## Abstract

**Background:**

Postoperative anemia is a common complication of metabolic and bariatric surgery (MBS), particularly among female patients. The predictive value of elevated preoperative red cell distribution width (RDW), a marker of erythropoietic stress, for incident postoperative anemia in nonanemic patients remains unclear.

**Methods:**

Data from the TriNetX Global Collaborative Network were used to classify adult female patients without preoperative anemia who underwent primary MBS between 2010 and 2024 into elevated RDW (>14.5%) and normal RDW (11.5–14.5%) groups. The primary outcome was incident anemia (hemoglobin <12 g/dL) over 1 year, assessed from a 30-day postoperative landmark to reduce perioperative bias. Secondary outcomes included incident iron deficiency anemia (IDA), non-IDA nutritional anemia, ferritin <30 ng/mL, subsequent RDW elevation, and hospital readmissions. Multivariable Cox regression was additionally performed in the unmatched cohort.

**Results:**

After matching, 8,809 pairs were analyzed. Incident anemia occurred in 17.9 and 13.6% of patients in the elevated and normal RDW groups (hazard ratio [HR] 1.35, 95% confidence interval [CI]: 1.25–1.45; *p* < 0.001), with an absolute risk difference of 4.3% (approximate number needed to screen, 23). Incident IDA was also higher in the elevated RDW group (HR 1.93, 95% CI: 1.64–2.28; *p* < 0.001). Ferritin <30 ng/mL (HR 1.51; *p* < 0.001) and subsequent RDW elevation (HR 3.38; *p* < 0.001) were significantly more frequent, whereas non-IDA nutritional anemia and hospital readmission did not differ. In multivariable analyses, elevated RDW remained independently associated with anemia (HR 1.60) and IDA (HR 2.13), with effect sizes comparable to those of ferritin <30 ng/mL. The associations were consistent over 3 years, across subgroups, and at a higher RDW threshold (>15%). In the smaller male cohort, elevated RDW was associated with iron-related markers (Ferritin <30 ng/mL) but not with incident anemia, possibly reflecting limited statistical power.

**Conclusion:**

Elevated preoperative RDW is independently associated with an increased risk of incident anemia and IDA in non-anemic female patients undergoing MBS. As a routinely available laboratory parameter, RDW may provide complementary value for preoperative risk stratification and identification of patients who may benefit from closer postoperative hematologic monitoring in the future.

## 1 Introduction

Metabolic and bariatric surgery (MBS) is the most effective treatment for sustained weight loss and improvement of obesity-related comorbidities, with hundreds of thousands of procedures performed worldwide annually (1–5). Despite its established benefits, postoperative anemia remains one of the most common nutritional complications, with reported rates ranging from approximately 15 to 40% during the first postoperative year (6–10). Female patients are disproportionately affected, likely owing to menstrual iron loss and lower baseline iron stores (11, 12), both of which may increase the vulnerability to postoperative iron deficiency and anemia. In addition, anatomical and physiological changes after Roux-en-Y gastric bypass and sleeve gastrectomy can impair iron absorption and increase the risk of postoperative anemia, particularly iron deficiency–related anemia (10, 13–15).

Red cell distribution width (RDW), a routinely reported parameter in complete blood count, reflects the degree of anisocytosis in circulating erythrocytes (16, 17). Elevated RDW may reflect early abnormalities in erythropoiesis related to iron deficiency, nutritional deficiencies, and inflammation and can be present before overt anemia is clinically apparent (16–19). This makes RDW a biologically plausible early indicator of MBS, where postoperative iron absorption is structurally compromised. Although elevated preoperative RDW has been linked to adverse postoperative outcomes (20–25), including mortality and renal dysfunction, its role as a predictor of incident postoperative anemia in MBS patients has not been established.

Previous studies have largely focused on preoperative anemia or iron deficiency as risk factors for postoperative anemia after MBS (8, 26). Therefore, risk stratification remains insufficiently defined for patients who undergo MBS with normal preoperative hemoglobin levels. Better identification of high-risk individuals within this large and frequently overlooked population would be clinically valuable, as it could support earlier intervention and more targeted nutritional optimization. To address this gap, we conducted a large propensity score-matched cohort study using the TriNetX Global Collaborative Network to examine whether an elevated preoperative RDW is associated with an increased risk of incident anemia and IDA in non-anemic female patients undergoing MBS.

## 2 Methods

### 2.1 Data sources

This retrospective cohort study was based on de-identified electronic health record data obtained from the TriNetX Global Collaborative Network, a federated real-world database comprising 171 healthcare organizations across multiple countries. The TriNetX database has been widely used in peer-reviewed clinical and epidemiological research (27–30). This study was approved by the Institutional Review Board of Chi Mei Medical Center, which waived the requirement for informed consent. All procedures were performed in accordance with the principles of the Declaration of Helsinki. Because the dataset was fully de-identified and compliant with the Health Insurance Portability and Accountability Act (HIPAA), individual patient consent was not required.

### 2.2 Exposure definition

Adult female patients (aged ≥18 years) who underwent MBS between January 1, 2010, and December 31, 2024, were eligible for inclusion if they had no evidence of preoperative anemia. Patients were excluded if they had a hemoglobin concentration ≤11.9 g/dL within 3 months prior to the index operation or a prior diagnosis of nutritional anemia, including iron deficiency anemia (IDA) (ICD-10-CM D50), vitamin B12 deficiency anemia (ICD-10-CM D51), folate deficiency anemia (ICD-10-CM D52), or other nutritional anemias (ICD-10-CM D53). Baseline exclusions were applied to prior nutritional anemia diagnoses to ensure that postoperative outcomes represented incident events after the 30-day landmark. Bariatric procedures were defined using the Current Procedural Terminology (CPT) codes for primary (first-time) laparoscopic Roux-en-Y gastric bypass or sleeve gastrectomy. The index date was defined as the date of the first qualifying MBS.

To ensure appropriate exposure classification, all included patients were required to have at least one preoperative red cell distribution width (RDW) measurement within 3 months prior to the index surgery. Among this non-anemic female cohort, patients were categorized into two groups based on RDW values: an elevated RDW group (>14.5%) and a normal RDW group (11.5–14.5%). Patients with overlapping or inconsistent RDW values across these ranges were excluded to maintain a clear exposure separation.

### 2.3 Exclusion criteria

Beyond the non-anemia eligibility requirement described above, patients with advanced chronic kidney disease (stage 4–5) or end-stage renal disease within 3 years prior to the index date were excluded due to their high baseline risk of anemia and altered erythropoiesis. Patients were also required to survive for at least 30 days following surgery to reduce immortal time bias and exclude perioperative mortality. The complete code-level definitions for all exclusion criteria are provided in Supplementary Table 1.

### 2.4 Data collection and propensity score matching

Baseline characteristics were extracted across four domains and used to estimate propensity scores: demographics (age, race, and BMI category), comorbidities (e.g., essential hypertension, diabetes mellitus, chronic kidney disease, neoplasms, alcohol-related disorders, and nicotine dependence), preoperative laboratory indices (ferritin < 30 ng/mL, albumin, HbA1c, vitamin B12, and C-reactive protein), and concomitant medications (e.g., GLP-1 receptor agonists, iron supplements). These variables were selected because of their established or biologically plausible roles as confounders of the RDW–anemia relationship, particularly those influencing systemic inflammation, iron stores, glycemic burden, and erythropoietic capacity. One-to-one propensity score matching was subsequently performed using a greedy nearest-neighbor algorithm constrained to a caliper of 0.1 standard deviation of the logit-transformed propensity score. In practical terms, this approach matched each patient with elevated RDW to a clinically similar patient with normal RDW based on measured baseline characteristics. The resulting covariate balance was quantified using standardized mean differences (SMDs), with an absolute SMD < 0.10 considered to indicate acceptable post-matching balance for measured covariates. The complete code-level definitions for all variables are provided in Supplementary Table 1.

### 2.5 Primary and secondary outcomes

The primary outcome was incident anemia, defined as a hemoglobin level < 12 g/dL, ascertained over a 1-year follow-up period starting from a 30-day landmark after the index date. This threshold was selected because hemoglobin <12 g/dL is a commonly used clinical definition of anemia in adult women and provides a standardized laboratory-based outcome definition across healthcare organizations. Secondary outcomes included incident IDA, non-IDA nutritional anemia, ferritin < 30 ng/mL, subsequent RDW elevation > 14.5%, and hospital readmission. We separated IDA from non-IDA nutritional anemia for clinical and analytical reasons. IDA was analyzed separately using ICD-10-CM D50 codes because it is the most frequent and clinically relevant nutritional anemia subtype after MBS and had sufficient event numbers for separate analysis. In contrast, vitamin B12 deficiency anemia, folate deficiency anemia, and other nutritional anemias (ICD-10-CM D51–D53) were individually less frequent and were therefore grouped as non-IDA nutritional anemia to preserve statistical power. This separation also allowed us to evaluate whether elevated preoperative RDW was preferentially associated with iron-related anemia rather than with nutritional anemia subtypes more broadly.

Ferritin < 30 ng/mL was included to capture subclinical iron depletion preceding overt anemia. Subsequent RDW elevation above 14.5% was assessed to determine whether preoperative erythropoietic stress persisted or worsened postoperatively, and hospital readmission was examined as a clinically meaningful indicator of postoperative morbidity burden. The incidence of hemoglobin measurement during follow-up was additionally assessed as a proxy for healthcare utilization and to evaluate the potential outcome ascertainment imbalance between groups. To evaluate whether the observed risk persisted over a longer horizon, the association between elevated RDW and incident anemia was examined over a 3-year follow-up period in the female cohort using the same matched population and analytic approach. All outcome definitions with corresponding diagnostic codes are detailed in Supplementary Table 1.

### 2.6 Subgroup and additional analysis

Subgroup analyses were conducted according to age (18–50 vs. >50 years) and surgical procedure type (sleeve gastrectomy vs. Roux-en-Y gastric bypass). The age strata were selected to partially address the possibility that menstrual status, menopausal status, and age-related differences in iron reserve could influence the association between RDW and postoperative anemia, although these factors were not directly captured in TriNetX. Within each stratum, propensity score matching and time-to-event analyses were independently repeated using the same framework as in the primary analysis, and formal interaction testing was performed to evaluate effect modification. A sensitivity analysis employing a more stringent RDW threshold (>15%) was conducted using the same normal RDW reference group, eligibility criteria, and statistical methods to examine whether a higher degree of RDW elevation carried an incrementally greater anemia risk. A parallel analysis in male patients was performed to assess the generalizability of the primary findings beyond the female cohort. In this analysis, incident anemia was defined as a hemoglobin level < 13 g/dL, consistent with the World Health Organization sex-specific threshold.

### 2.7 Statistical analysis

Time-to-event analyses were performed using Cox proportional hazards regression, and the findings are reported as hazard ratios (HRs) and 95% confidence intervals (CIs). The proportional hazards assumptions were verified for all models using Schoenfeld residual testing. To complement the matched analysis, a multivariable Cox regression model was fitted in the unmatched cohort to assess the independent association of elevated RDW with incident anemia and IDA after adjusting for pre-specified covariates. For the primary outcome, E-values were calculated to quantify the minimum association strength that an unmeasured confounder would require to fully account for the observed effect estimate, without implying that residual confounding was removed, adjusted for, or corrected. Statistical significance for the primary analysis was set at a two-tailed *p*-value of < 0.05. Secondary outcomes, subgroup analyses, sensitivity analyses, and design validation metrics were treated as exploratory and interpreted without correction for multiple comparisons. All analyses were performed using the TriNetX analytics platform. Missing data were handled as recorded in the source records without imputation. Because all analyses were conducted within the TriNetX analytics environment, competing risk analysis for death before incident anemia was not feasible due to platform constraints.

## 3 Results

### 3.1 Baseline characteristics and primary outcomes

Prior to propensity score matching, 8,863 female patients with elevated preoperative RDW (>14.5%) and 34,118 with normal RDW (11.5–14.5%) were identified. After matching, 8,809 pairs were retained. The mean age after matching was 44.3 ± 11.9 years in the elevated RDW group and 44.1 ± 11.9 years in the control group. All standardized mean differences (SMDs) for the baseline covariates fell below 0.10 following matching, indicating adequate covariate balance across demographic characteristics, comorbidities, preoperative laboratory indices, and concomitant medications (Table 1).

**Table 1:** Baseline characteristics of non-anemic female patients undergoing metabolic and bariatric surgery, stratified by preoperative red cell distribution width before and after propensity score matching.

Variables	Before matching			After matching		
	Elevated RDW group (*n* = 8,863)	Control group (*n* = 34,118)	SMD	Elevated RDW group (*n* = 8,809)	Control group (*n* = 8,809)	SMD
Patient characteristics						
Age at index (years)	44.4 ± 11.9	42.7 ± 11.8	0.135	44.3 ± 11.9	44.1 ± 11.9	0.018
BMI 35–39.9 (kg/m^2^)	2,218 (25.0)	11,627 (34.1)	0.199	2,213 (25.1)	2,147 (24.4)	0.017
BMI 40–44.9 (kg/m^2^)	3,553 (40.1)	16,287 (47.7)	0.155	3,545 (40.2)	3,394 (38.5)	0.035
BMI 45–50 (kg/m^2^)	3,165 (35.7)	11,712 (34.3)	0.029	3,144 (35.7)	3,109 (35.3)	0.008
White	4,793 (54.1)	23,861 (69.9)	0.331	4,791 (54.4)	4,776 (54.2)	0.003
Black or African American	2,952 (33.3)	5,908 (17.3)	0.374	2,904 (33.0)	2,950 (33.5)	0.011
Asian	52 (0.6)	177 (0.5)	0.009	52 (0.6)	44 (0.5)	0.012
Comorbidities						
Essential hypertension	5,571 (62.9)	18,187 (53.3)	0.194	5,527 (62.7)	5,515 (62.6)	0.003
Sleep apnea	5,413 (61.1)	18,437 (54.0)	0.143	5,375 (61.0)	5,365 (60.9)	0.002
Other anxiety disorders	3,515 (39.7)	14,583 (42.7)	0.063	3,494 (39.7)	3,542 (40.2)	0.011
Diabetes mellitus	3,287 (37.1)	9,525 (27.9)	0.197	3,247 (36.9)	3,154 (35.8)	0.022
Diseases of liver	2,118 (23.9)	8,535 (25.0)	0.026	2,110 (24.0)	2090 (23.7)	0.005
Neoplasms	1,698 (19.2)	6,190 (18.1)	0.026	1,688 (19.2)	1,653 (18.8)	0.010
Major depressive disorder	955 (10.8)	3,779 (11.1)	0.010	947 (10.8)	950 (10.8)	0.001
Nicotine dependence	854 (9.6)	3,509 (10.3)	0.022	853 (9.7)	824 (9.4)	0.011
Ischemic heart diseases	695 (7.8)	1833 (5.4)	0.100	688 (7.8)	680 (7.7)	0.003
COPD	463 (5.2)	1,088 (3.2)	0.102	453 (5.1)	421 (4.8)	0.017
CKD	323 (3.6)	798 (2.3)	0.077	320 (3.6)	318 (3.6)	0.001
Cerebrovascular diseases	233 (2.6)	615 (1.8)	0.056	230 (2.6)	222 (2.5)	0.006
Alcohol related disorders	143 (1.6)	534 (1.6)	0.004	141 (1.6)	135 (1.5)	0.005
Laboratory data						
Albumin ≥ 3.5 g/dL	7,814 (88.2)	30,646 (89.8)	0.053	7,773 (88.2)	7,803 (88.6)	0.011
HbA1c ≥ 7%	1,325 (15.0)	3,574 (10.5)	0.135	1,307 (14.8)	1,280 (14.5)	0.009
Vitamin B12 ≤ 200 pg./mL	174 (2.0)	548 (1.6)	0.027	173 (2.0)	158 (1.8)	0.013
Ferritin ≤ 30 ng/mL	1,564 (17.6)	3,129 (9.2)	0.251	1,521 (17.3)	1,498 (17.0)	0.007
C-reactive protein ≥ 10 mg/L	693 (7.8)	2039 (6.0)	0.073	684 (7.8)	680 (7.7)	0.002
Medications						
Insulins and analogues	2,439 (27.5)	7,273 (21.3)	0.145	2,411 (27.4)	2,340 (26.6)	0.018
Biguanides	1937 (21.9)	6,110 (17.9)	0.099	1913 (21.7)	1864 (21.2)	0.014
GLP-1 analogues	1,138 (12.8)	3,951 (11.6)	0.038	1,124 (12.8)	1,077 (12.2)	0.016
Iron supplementation	1,096 (12.4)	2,608 (7.6)	0.158	1,075 (12.2)	1,060 (12.0)	0.005
Sulfonylureas	354 (4.0)	954 (2.8)	0.066	346 (3.9)	329 (3.7)	0.010
SGLT2 inhibitors	305 (3.4)	790 (2.3)	0.067	301 (3.4)	284 (3.2)	0.011
DPP-4 inhibitors	217 (2.4)	606 (1.8)	0.047	212 (2.4)	209 (2.4)	0.002
Type of surgery						
Sleeve surgery	6,219 (70.2)	22,512 (66.0)	0.090	6,177 (70.1)	6,212 (70.5)	0.009
Bypass surgery	2,693 (30.4)	11,725 (34.4)	0.085	2,680 (30.4)	2,647 (30.0)	0.008

Data are presented as *n* (%) for categorical variables and mean ± standard deviation for continuous variables. Standardized mean differences (SMDs) were used to assess covariate balance, with an absolute SMD of <0.10 indicating adequate balance. RDW, red cell distribution width; BMI, body mass index; CKD, chronic kidney disease; COPD, chronic obstructive pulmonary disease; HbA1c, hemoglobin A1c; GLP-1, glucagon-like peptide-1; SGLT2, sodium–glucose cotransporter 2; DPP-4: Dipeptidyl peptidase 4.

Over the 1-year follow-up period, incident anemia (hemoglobin <12 g/dL) occurred in 1,578 patients (17.9%) in the elevated RDW group compared with 1,201 patients (13.6%) in the control group (HR 1.35, 95% CI: 1.25–1.45; *p* < 0.001) (Table 2). The absolute risk difference was 4.3%, corresponding to an approximate number needed to screen of 23 patients with elevated RDW to identify one additional case of postoperative anemia compared with patients with normal RDW. Incident IDA was observed in 4.8% versus 2.5% of patients, respectively (HR 1.93, 95% CI: 1.64–2.28; *p* < 0.001). The E-value for the primary point estimate was 1.76 (95% CI lower bound: 1.61), indicating that an unmeasured confounder would need to be associated with both elevated RDW and anemia risk by a factor of at least 1.76 to fully explain the observed association. Because this E-value was modest, residual confounding cannot be excluded. Non-IDA nutritional anemia was not significantly different between groups (HR 1.16, *p* = 0.223). Ferritin < 30 ng/mL (HR 1.51, *p* < 0.001) and subsequent RDW elevation > 14.5% (HR 3.38, *p* < 0.001) occurred more frequently in the elevated RDW group, whereas hospital readmission did not differ significantly (HR 1.03, *p* = 0.528).

**Table 2:** Association between elevated preoperative red cell distribution width and the 1-year risk of incident anemia and related outcomes.

Outcome	Elevated RDW group (*n* = 8,809)	Control group (*n* = 8,809)	HR (95% CI)	*p*-value
	Events (%)	Events (%)		
Anemia (Hb < 12 g/dL)	1,578 (17.9)	1,201 (13.6)	1.35 (1.25–1.45)	<0.001
IDA	420 (4.8)	219 (2.5)	1.93 (1.64–2.28)	<0.001
Non-IDA nutritional anemia^‡^	140 (1.6)	120 (1.4)	1.16 (0.91–1.49)	0.223
Ferritin <30 ng/mL	1,338 (15.2)	910 (10.3)	1.51 (1.38–1.64)	<0.001
RDW > 14.5%	4,640 (52.7)	1,698 (19.3)	3.38 (3.20–3.58)	<0.001
Hospital readmission	1,107 (12.6)	1,077 (12.2)	1.03 (0.95–1.12)	0.528
Follow-up Hb measurement	6,025 (68.4)	6,142 (69.7)	0.96 (0.93–1.00)	0.031

RDW, red cell distribution width; IDA, iron deficiency anemia; CI, confidence interval; HR, hazard ratio; Hb: hemoglobin; ^‡^Non-IDA nutritional anemia included vitamin B12 deficiency anemia, folate deficiency anemia, and other nutritional anemias.

Over the 3-year follow-up, similar patterns were observed for incident anemia (HR 1.30, *p* < 0.001), IDA (HR 1.75, *p* < 0.001), ferritin < 30 ng/mL (HR 1.35, *p* < 0.001), and subsequent RDW elevation (HR 3.24, *p* < 0.001), while non-IDA nutritional anemia (HR 1.15, *p* = 0.137) and hospital readmission remained non-significant (HR 0.99, *p* = 0.851) (Table 3).

**Table 3:** Association between elevated preoperative red cell distribution width and the 3-year risk of incident anemia and related outcomes.

Outcome	Elevated RDW group (*n* = 8,809)	Control group (*n* = 8,809)	HR (95% CI)	*p-*value
	Events (%)	Events (%)		
Anemia (Hb < 12 g/dL)	2,913 (33.1)	2,325 (26.4)	1.30 (1.23–1.38)	<0.001
IDA	1,057 (12.0)	613 (7.0)	1.75 (1.59–1.94)	<0.001
Non-IDA nutritional anemia^‡^	247 (2.8)	213 (2.4)	1.15 (0.96–1.38)	0.137
Ferritin <30 ng/mL	2,156 (24.5)	1,657 (18.8)	1.35 (1.26–1.44)	<0.001
RDW > 14.5%	5,426 (61.6)	2,248 (25.5)	3.24 (3.08–3.40)	<0.001
Hospital readmission	2,118 (24.0)	2,108 (23.9)	0.99 (0.94–1.06)	0.851
Follow-up Hb measurement	6,957 (79.0)	7,027 (79.8)	0.96 (0.93–0.99)	0.019

RDW, red cell distribution width; IDA, iron deficiency anemia; CI, confidence interval; HR, hazard ratio; Hb: hemoglobin; ^‡^Non-IDA nutritional anemia included vitamin B12 deficiency anemia, folate deficiency anemia, and other nutritional anemias.

### 3.2 Sensitivity analysis with a higher RDW threshold

When the RDW exposure threshold was raised to >15%, a matched cohort of 5,964 pairs was analyzed using the same method. The associations with incident anemia (HR 1.35, *p* < 0.001) and IDA (HR 1.84, *p* < 0.001) remained statistically significant and comparable to the primary analysis. Ferritin levels < 30 ng/mL (HR 1.47, *p* < 0.001) and subsequent RDW elevation (HR 3.52, *p* < 0.001) were similarly elevated in the higher-threshold group, whereas non-IDA nutritional anemia (*p* = 0.345) and hospital readmission (*p* = 0.547) were not significantly different between the groups (Table 4).

**Table 4:** Sensitivity analysis: association between elevated preoperative red cell distribution width (>15%) and the 1-year risk of incident anemia and related outcomes.

Outcome	Elevated RDW group (*n* = 5,964)	Control group (*n* = 5,964)	HR (95% CI)	*p-*value
	Events (%)	Events (%)		
Anemia (Hb < 12 g/dL)	1,125 (18.9)	853 (14.3)	1.35 (1.24–1.48)	<0.001
IDA	302 (5.1)	166 (2.8)	1.84 (1.52–2.22)	<0.001
Non-IDA nutritional anemia^‡^	100 (1.7)	87 (1.5)	1.15 (0.86–1.53)	0.345
Ferritin <30 ng/mL	943 (15.8)	659 (11.1)	1.47 (1.33–1.62)	<0.001
RDW > 14.5%	3,307 (55.4)	1,190 (20.0)	3.52 (3.29–3.76)	<0.001
Hospital readmission	744 (12.5)	723 (12.1)	1.03 (0.93–1.14)	0.547
Follow-up Hb measurement	4,051 (67.9)	4,182 (70.1)	0.94 (0.90–0.98)	0.003

RDW, red cell distribution width; IDA, iron deficiency anemia; CI, confidence interval; HR, hazard ratio; Hb: hemoglobin; ^‡^Non-IDA nutritional anemia included vitamin B12 deficiency anemia, folate deficiency anemia, and other nutritional anemias.

### 3.3 Subgroup and parallel analyses

Subgroup analyses by age demonstrated associations between elevated RDW and incident anemia in both the 18–50 years stratum (HR 1.46, *p* < 0.001) and >50 years stratum (HR 1.33, *p* < 0.001), with no significant interaction (p for interaction = 0.230). Associations with IDA were also present in both age groups (HR 1.92 and 1.55, respectively; p for interaction = 0.185) (Table 5).

**Table 5:** Subgroup analysis by age for the association between elevated preoperative red cell distribution width and the 1-year risk of anemia.

Outcomes	18–50 yrs.^†^		>50 yrs.^¶^		p for interaction
	HR (95% CI)	*p*-value	HR (95% CI)	*p-*value	
Anemia (Hb < 12 g/dL)	1.46 (1.31–1.62)	<0.001	1.33 (1.19–1.48)	<0.001	0.230
IDA	1.92 (1.54–2.39)	<0.001	1.55 (1.24–1.93)	<0.001	0.185
Non-IDA nutritional anemia^‡^	1.18 (0.81–1.72)	0.397	1.08 (0.79–1.48)	0.620	0.731
Ferritin <30 ng/mL	1.41 (1.27–1.57)	<0.001	1.61 (1.39–1.85)	<0.001	0.153
RDW > 14.5%	3.38 (3.11–3.67)	<0.001	3.24 (3.01–3.49)	<0.001	0.457
Hospital readmission	1.05 (0.93–1.19)	0.403	1.00 (0.89–1.13)	0.980	0.580
Follow-up Hb measurement	0.92 (0.88–0.97)	0.001	0.98 (0.93–1.03)	0.330	0.080

^†^*n* = 4,393 per group; *^¶^n* = 4,401 per group; RDW, red cell distribution width; IDA, iron deficiency anemia; CI, confidence interval; HR, hazard ratio; Hb: hemoglobin; ^‡^Non-IDA nutritional anemia included vitamin B12 deficiency anemia, folate deficiency anemia, and other nutritional anemias.

When stratified by surgical procedure, an elevated RDW was associated with incident anemia in both the sleeve gastrectomy (HR 1.32, *p* < 0.001) and Roux-en-Y gastric bypass (HR 1.41, *p* < 0.001) subgroups, with no significant interaction (*p* = 0.401). A significant interaction by procedure type was detected for subsequent RDW elevation above 14.5% (p for interaction = 0.001), with a numerically higher HR observed in the sleeve gastrectomy subgroup (HR 3.61 vs. 3.01) (Table 6).

**Table 6:** Subgroup analysis by surgical procedure for the association between elevated preoperative red cell distribution width and the 1-year risk of anemia.

Outcomes	Sleeve surgery^†^		Bypass surgery^¶^		p for interaction
	HR (95% CI)	*p*-value	HR (95% CI)	*p-*value	
Anemia (Hb < 12 g/dL)	1.32 (1.20–1.46)	<0.001	1.41 (1.25–1.58)	<0.001	0.401
IDA	1.81 (1.46–2.25)	<0.001	2.30 (1.79–2.96)	<0.001	0.174
Non-IDA nutritional anemia^‡^	0.96 (0.70–1.31)	0.790	1.23 (0.86–1.77)	0.264	0.334
Ferritin <30 ng/mL	1.45 (1.30–1.62)	<0.001	1.38 (1.21–1.56)	<0.001	0.563
RDW > 14.5%	3.61 (3.36–3.87)	<0.001	3.01 (2.75–3.28)	<0.001	0.001
Hospital readmission	0.97 (0.88–1.07)	0.520	1.01 (0.87–1.17)	0.899	0.659
Follow-up Hb measurement	0.94 (0.90–0.98)	0.010	0.94 (0.88–1.00)	0.040	1.000

^†^*n* = 6,155 per group; *^¶^n* = 2,769 per group; RDW, red cell distribution width; IDA, iron deficiency anemia; CI, confidence interval; HR, hazard ratio; Hb: hemoglobin; ^‡^Non-IDA nutritional anemia included vitamin B12 deficiency anemia, folate deficiency anemia, and other nutritional anemias.

A parallel analysis in male patients (n = 1,638 matched pairs) found no statistically significant association between elevated RDW and incident anemia (HR 1.16, *p* = 0.125) or IDA (HR 1.03, *p* = 0.899) in male patients. Ferritin < 30 ng/mL (HR 2.17, *p* < 0.001), subsequent RDW elevation (HR 3.38, *p* < 0.001), and hospital readmission (HR 1.26, *p* = 0.027) were significantly higher in the elevated RDW group (Table 7).

**Table 7:** Association between elevated preoperative red cell distribution width and the 1-year risk of incident anemia and related outcomes in male patients.

Outcome	Elevated RDW group (*n* = 1,638)	Control group (*n* = 1,638)	HR (95% CI)	*p-*value
	Events (%)	Events (%)		
Anemia (Hb < 13 g/dL)	236 (14.4)	202 (12.3)	1.16 (0.96–1.40)	0.125
IDA	46 (2.8)	44 (2.7)	1.03 (0.68–1.55)	0.899
Non-IDA nutritional anemia^‡^	15 (0.9)	20 (1.2)	0.73 (0.38–1.43)	0.364
Ferritin <30 ng/mL	92 (5.6)	42 (2.6)	2.17 (1.51–3.13)	<0.001
RDW > 14.5%	848 (51.8)	306 (18.7)	3.38 (2.97–3.86)	<0.001
Hospital readmission	205 (12.5)	162 (9.9)	1.26 (1.03–1.55)	0.027
Follow-up Hb measurement	1,119 (68.3)	1,066 (65.1)	1.03 (0.94–1.12)	0.558

RDW, red cell distribution width; IDA, iron deficiency anemia; CI, confidence interval; HR, hazard ratio; Hb: hemoglobin; ^‡^Non-IDA nutritional anemia included vitamin B12 deficiency anemia, folate deficiency anemia, and other nutritional anemias.

### 3.4 Multivariable Cox regression analysis

**Figure 1:**
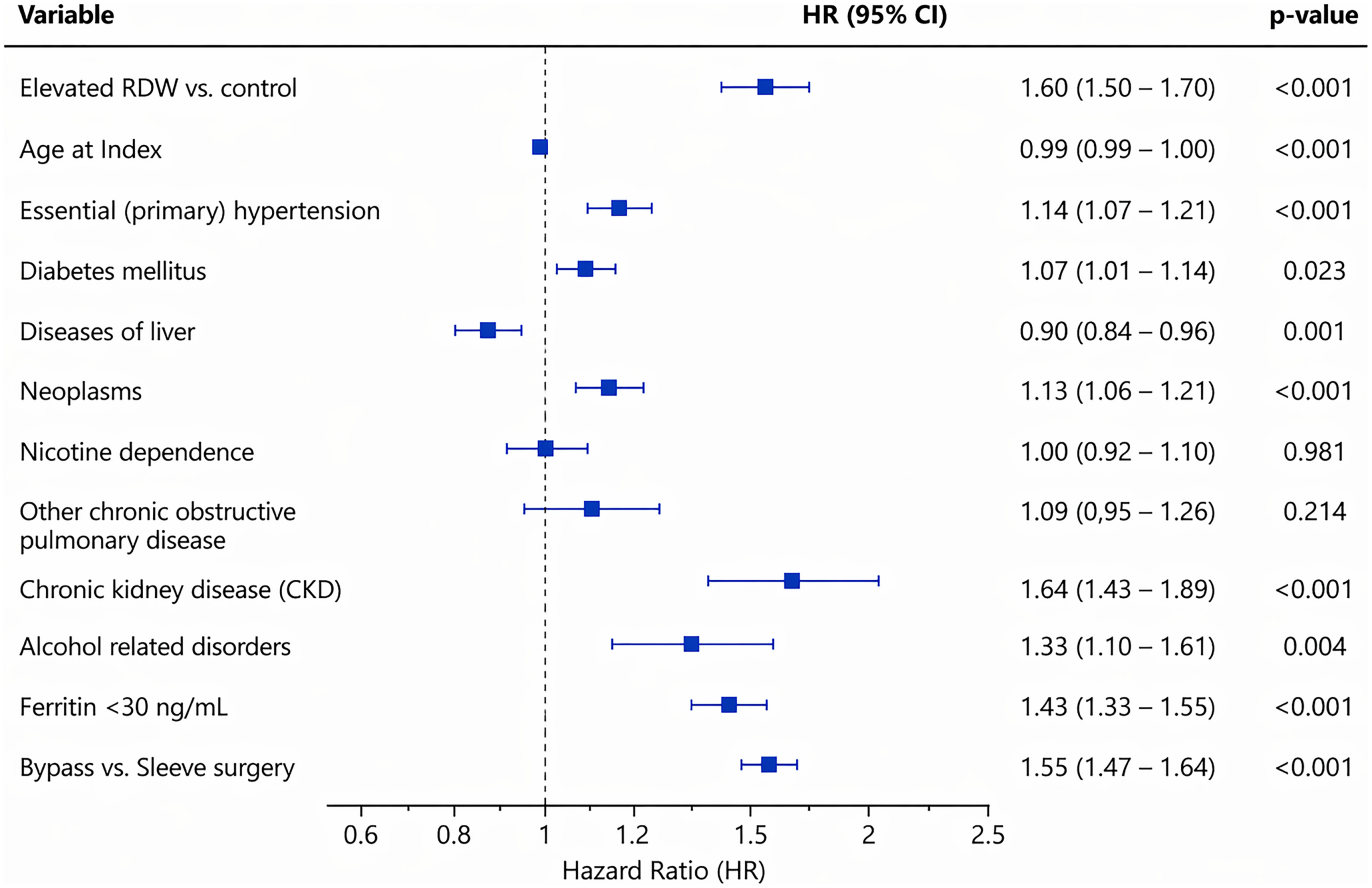
Multivariable Cox proportional hazards regression analysis for incident anemia at 1-year follow-up in the unmatched cohort. RDW, red cell distribution width; HR, hazard ratio; CI, confidence interval.

**Figure 2:**
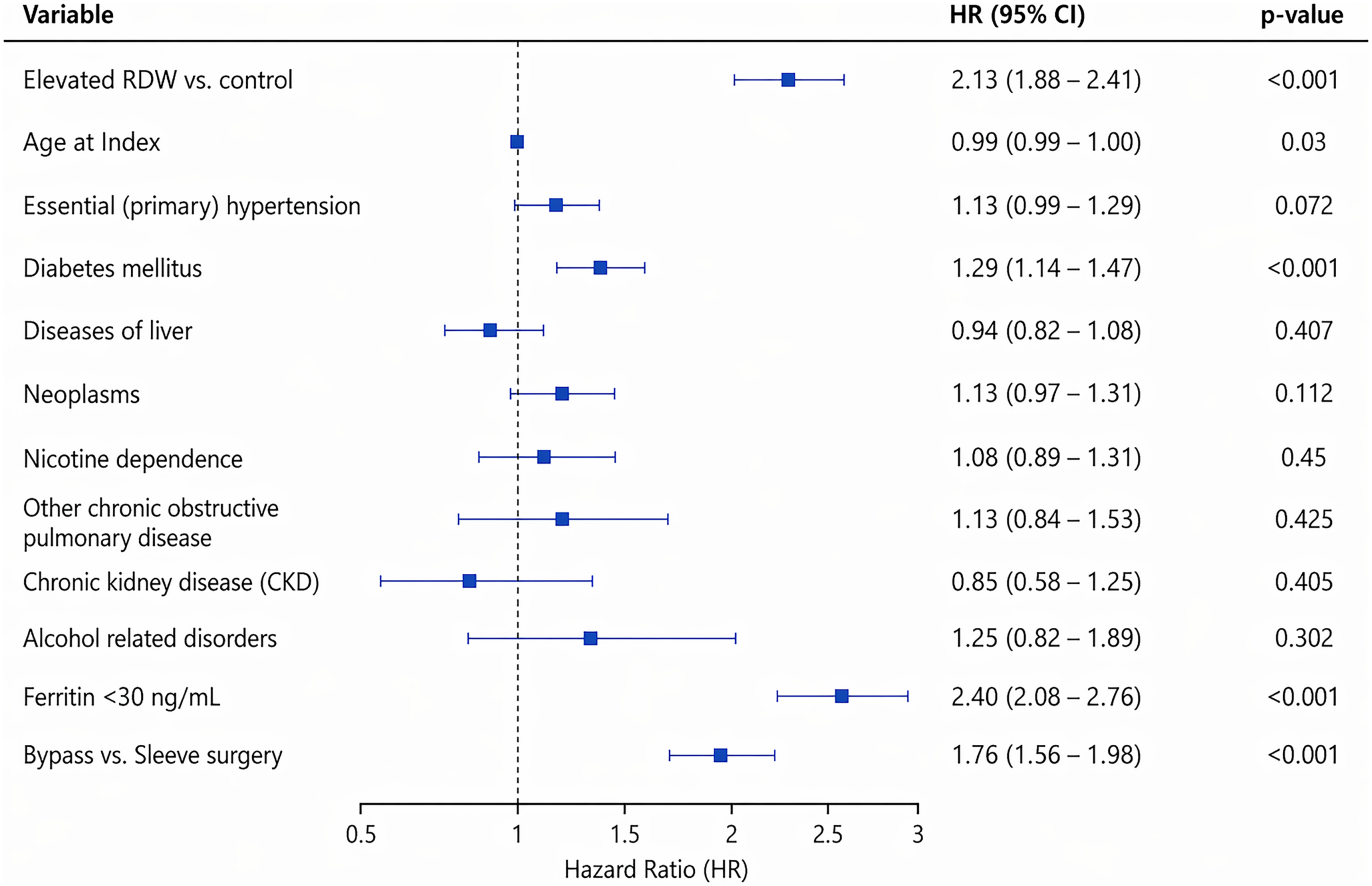
Multivariable Cox proportional hazards regression analysis for incident iron deficiency anemia (IDA) at 1-year follow-up in the unmatched cohort. RDW, red cell distribution width; IDA, iron deficiency anemia; HR, hazard ratio; CI, confidence interval.

In the unmatched cohort, multivariable Cox regression confirmed that an elevated RDW was independently associated with both incident anemia (HR 1.60, *p* < 0.001) and incident IDA (HR 2.13, *p* < 0.001) at 1 year (Figures 1, 2). Additional covariates independently associated with incident anemia included CKD (HR 1.64, *p* < 0.001), bypass versus sleeve surgery (HR 1.55, *p* < 0.001), ferritin < 30 ng/mL (HR 1.43, *p* < 0.001), and alcohol-related disorders (HR 1.33, *p* = 0.004). For incident IDA, elevated RDW (HR 2.13) and ferritin levels < 30 ng/mL (HR 2.40) represented the two strongest independent associations (both *p* < 0.001), followed by bypass surgery (HR 1.76, *p* < 0.001).

## 4 Discussion

In this large propensity score-matched cohort study of non-anemic female patients undergoing MBS, elevated preoperative RDW was independently associated with a higher risk of incident anemia and IDA over 1 year. These associations persisted over the 3-year follow-up, remained consistent across sensitivity and subgroup analyses, and were confirmed using multivariable Cox regression in the unmatched cohort. Notably, no comparable association was observed in the parallel male analysis, which may reflect a potential sex-specific difference, although this finding should be interpreted cautiously, given the smaller male sample size. The estimated number needed to screen was approximately 23, indicating that RDW should be interpreted as an adjunctive risk marker rather than a stand-alone clinical decision tool.

The biological plausibility of these findings may be considered in relation to RDW as an integrated marker of erythropoietic stress. Elevated RDW reflects greater heterogeneity in circulating red blood cell size and has been associated with subclinical iron depletion, early nutritional deficiencies, and chronic low-grade inflammation, all of which may precede the onset of overt anemia (16–19). In patients undergoing MBS, postoperative iron absorption may be altered by anatomical and physiological changes in the gastrointestinal tract. Accordingly, patients with higher preoperative RDW may represent a subgroup with a reduced hematologic reserve and may therefore be more susceptible to developing postoperative anemia. From this perspective, an elevated preoperative RDW may reflect an underlying nutritional vulnerability that becomes more clinically apparent during the postoperative period. Because RDW may be elevated in iron deficiency, vitamin B12 or folate deficiency, inflammation, and other forms of disordered erythropoiesis, we separately analyzed IDA and non-IDA nutritional anemia to avoid interpreting RDW as an iron-specific marker.

Prior studies have consistently shown that an elevated RDW is associated with adverse surgical outcomes, including higher mortality after noncardiac surgery (21, 22), acute kidney injury after cardiac surgery (23), and worse prognosis following hip fracture (24). However, these investigations have primarily involved cardiovascular or orthopedic populations, and the association between RDW and postoperative hematologic outcomes after MBS has not been thoroughly examined. Previous studies in patients undergoing MBS have reported associations between preoperative anemia, iron deficiency, and an increased risk of postoperative anemia (8, 26); however, they have not specifically addressed the larger group of patients who undergo surgery with apparently normal hemoglobin levels. Our findings expand the potential prognostic role of RDW in this setting and suggest that it may help identify patients at increased risk within a cohort that would otherwise be considered to be at relatively low risk.

Several secondary and sensitivity analyses provided supportive evidence for the consistency of primary findings. The elevated risk of postoperative ferritin < 30 ng/mL (HR 1.51) in the elevated RDW group is consistent with the possibility that higher preoperative RDW identifies patients with greater susceptibility to postoperative iron depletion. The increased incidence of subsequent RDW elevation above 14.5% (HR 3.38) further suggests that the abnormality captured by RDW may persist beyond the preoperative period, although the underlying mechanisms cannot be determined from this study. When the exposure threshold was increased from >14.5 to >15.0%, the associations with anemia (HR 1.35) and IDA (HR 1.84) remained similar in magnitude, indicating that the observed association was robust to modest changes in exposure definition and was not driven by patients with borderline RDW values. The persistence of the association over 3 years further supports the maintenance of the observed relationship over a longer follow-up period.

Subgroup analyses demonstrated consistent associations across age groups and surgical procedure types, with no significant interactions with the primary outcome. Nevertheless, bariatric procedures may contribute to postoperative anemia through several mechanisms, including perioperative blood loss, reduced dietary intake, altered gastric acidity, impaired iron absorption, reduced intrinsic factor-related vitamin B12 absorption, and impaired absorption of other micronutrients. These mechanisms may differ between sleeve gastrectomy and Roux-en-Y gastric bypass, with bypass procedures generally producing greater malabsorptive effects, whereas sleeve gastrectomy may still affect gastric acidity, intake, and micronutrient tolerance. The absence of effect modification may suggest the relative homogeneity of the association across clinically relevant subgroups and supports the external validity of the findings within routine bariatric practice. However, the subgroup results do not exclude the possibility that procedure-specific mechanisms, perioperative blood loss, supplementation adherence, or postoperative monitoring contributed to the observed associations. These factors may be particularly relevant in female patients because menstrual iron losses and lower baseline iron stores can reduce hematologic reserve. A significant interaction was observed for subsequent RDW elevation according to procedure type, with a numerically higher hazard in the sleeve gastrectomy group than in the bypass group. This finding is hypothesis-generating and may reflect differences in postoperative care patterns or residual confounding, rather than a true biological effect.

The absence of a significant association between elevated RDW and incident anemia in men warrants careful interpretation. One possible explanation is the substantially smaller size of the male cohort (1,638 matched pairs vs. 8,809 matched pairs in females), which may have limited the statistical power to detect a modest association. Therefore, the male analysis should be interpreted as underpowered and inconclusive rather than as evidence that elevated RDW is not associated with postoperative anemia in men. Further studies with larger male cohorts are warranted. In addition to sample size considerations, potential sex-related biological differences may have contributed to this pattern. Female patients are generally more susceptible to postoperative iron deficiency because of ongoing menstrual blood loss and lower baseline iron reserves (10, 12), factors that may increase their vulnerability to hematologic disturbances after MBS. From this perspective, an elevated RDW in female patients may reflect a subgroup with a reduced hematologic reserve and greater susceptibility to postoperative anemia. However, this interpretation remains speculative, and the present study was not designed to directly determine the mechanisms underlying the observed sex-related differences.

From a clinical perspective, these findings suggest that preoperative RDW, a universally available, inexpensive, and routinely reported laboratory parameter, may be valuable for risk stratification of postoperative anemia in female patients undergoing MBS. Notably, in the multivariable Cox models, the magnitude of association for elevated RDW was comparable to that of ferritin < 30 ng/mL for both incident anemia (HR 1.60 vs. 1.43) and IDA (HR 2.13 vs. 2.40), supporting the potential utility of RDW as an accessible hematologic marker in this setting. Rather than replacing ferritin assessment, RDW may offer complementary information for identifying patients who could benefit from closer hematologic monitoring, further evaluation of iron status, and more individualized perioperative nutritional management. Given that anemia affects a substantial proportion of MBS patients and is associated with fatigue, impaired functional recovery, and increased healthcare utilization (31–33), earlier recognition of higher-risk patients may be clinically important.

Clinically, RDW should be viewed as an adjunctive risk signal rather than a stand-alone decision tool. In current bariatric pathways, an elevated preoperative RDW could prompt clinicians to review iron indices, ferritin, vitamin B12, folate, inflammatory status, and bleeding history more carefully before surgery. It may also help identify patients who warrant reinforced supplementation counseling and scheduled postoperative hemoglobin and micronutrient monitoring. However, our findings do not establish that RDW-guided intervention improves outcomes; therefore, RDW should complement, not replace, established nutritional assessment and follow-up protocols.

This study has several limitations. First, its observational design precludes causal inference, and residual confounding from unmeasured factors cannot be fully excluded. The E-value of 1.76 should be interpreted only as a quantitative sensitivity metric indicating the strength of unmeasured confounding that would be required to explain away the primary association; it does not remove or correct residual confounding. Accordingly, the findings should be interpreted as observational associations rather than evidence of a causal or definitive predictive relationship between RDW and postoperative anemia. Second, reliance on ICD-coded diagnoses may have introduced outcome misclassification, and the lack of additional hematologic parameters, such as mean corpuscular volume, transferrin saturation, and reticulocyte count, limited more detailed characterization of anemia subtypes. Inherited hematologic disorders, including hemoglobinopathies, thalassemias, and other inherited red cell disorders, were not specifically excluded or matched. Although not commonly emphasized in bariatric anemia risk stratification, these conditions may affect red cell indices and RDW interpretation; therefore, residual confounding or misclassification cannot be excluded. In addition, incident anemia was defined using a single recorded hemoglobin value below 12 g/dL, which may be affected by biological variability, transient clinical conditions, hydration status, or laboratory measurement error, thereby introducing potential outcome misclassification. Furthermore, although all patients were non-anemic at baseline and were matched on key determinants of hematologic reserve (including ferritin, vitamin B12, albumin, and C-reactive protein), baseline hemoglobin level itself was not included as a matching covariate or reported. Patients with elevated RDW may therefore have had lower baseline hemoglobin values, positioning them closer to the diagnostic threshold and contributing to their apparent risk of incident anemia during follow-up. This potential reverse causation, or residual confounding by baseline hematologic status, cannot be fully excluded and should be considered when interpreting the observed association.

Third, the TriNetX platform does not capture several potentially relevant clinical variables, including detailed surgical technique, perioperative blood loss, menopausal status, menstrual bleeding patterns, gynecologic causes of blood loss, adherence to postoperative supplementation, dietary intake, or intensity of postoperative laboratory monitoring. Therefore, these unmeasured procedure-related, nutritional, gynecologic, and surveillance factors may have contributed to the observed associations. Fourth, follow-up hemoglobin testing rates were broadly similar between the groups (HR 0.96), suggesting that major differential outcome ascertainment was unlikely. However, because testing rates differed slightly but significantly and not all patients underwent follow-up laboratory testing, residual outcome ascertainment bias cannot be excluded. Finally, because all analyses were conducted within the TriNetX analytics platform, competing risk analysis was not feasible due to platform constraints. The most relevant competing event was death before incident anemia, because patients who died during follow-up could no longer experience or have documented anemia outcomes. In addition, the 30-day survival eligibility criterion was used to establish a postoperative landmark cohort and reduce perioperative bias, but it may have excluded early postoperative deaths and thereby influenced interpretation of the observed associations.

## 5 Conclusion

Elevated preoperative RDW was associated with a higher risk of incident anemia and IDA in non-anemic female patients undergoing MBS, and these associations remained evident over 3 years. As a routinely available laboratory parameter, RDW may provide adjunctive value in preoperative risk assessment and may help inform consideration of closer nutritional and hematologic follow-up. Further prospective studies are needed to validate these findings and determine whether RDW-guided risk stratification can improve clinical outcomes.

## Data Availability

The raw data supporting the conclusions of this article will be made available by the authors, without undue reservation.
