# Supplementary material for: Elevated preoperative red cell distribution width and incident anemia after metabolic and bariatric surgery: a cohort study
Source: Front Nutr. 2026 Jul 13;13:1863689. doi: 10.3389/fnut.2026.1863689 (PMC13402206; doi:10.3389/fnut.2026.1863689)
Supplement: Supplementary file 1 [file Table_1.DOCX]

**Supplemental Table 1. Codes Used for Cohort Definition, Inclusion/Exclusion Criteria, Outcome Definitions, and Variables for Propensity Score Matching**

| Category | Variable | Definition / Code |
| --- | --- | --- |
| Exposure | High RDW | RDW ≥14.6% (TNX:9008) within 3 months before index |
|  | Control group | RDW 11.5–14.5% (TNX:9008), no RDW ≥14.6% |
| Index event | Bariatric surgery | CPT 43644 (RYGB), CPT 43775 (Sleeve gastrectomy) |
| Inclusion criteria | Age | ≥18 years |
|  | Sex | Female |
|  | Surgery period | 2010–2024 |
|  | RDW measurement | Within 3 months before surgery |
| Exclusion criteria | Advanced CKD | ICD-10: N18.4–N18.6 |
|  | Pre-existing anemia | ICD-10: D50–D53 |
|  | Preoperative anemia | Hb ≤11.9 g/dL (TNX:9014) |
|  | Early mortality | Death or R99 within 1 month after index |
| Primary outcome | Anemia | Hb ≤11.9 g/dL (TNX:9014) |
| Secondary outcomes | Iron deficiency anemia (IDA) | ICD-10: D50 |
|  | Other nutritional anemia | ICD-10: D51–D53 |
|  | Iron deficiency | Ferritin <30 ng/mL (TNX:9042) |
|  | RDW elevation | RDW ≥14.6% (TNX:9008) |
|  | Readmission | Inpatient encounter (HL7V3: IMP / NONAC / ACUTE) |
|  | Hb measurement | Any hemoglobin test (TNX:9014) |
| Matching variables | Demographics | Age, race |
|  | Comorbidities | Hypertension (I10), diabetes (E08–E13), CKD (N18), ischemic heart disease (I20–I25), COPD (J44), cerebrovascular disease (I60–I69), liver disease (K70–K77), sleep apnea (G47.3), depression (F33), anxiety (F41), nicotine dependence (F17), alcohol-related disorders (F10), neoplasms (C00–D49) |
|  | Procedures | RYGB (43644), sleeve gastrectomy (43775) |
|  | Medications | Insulin (A10A), metformin (A10BA), sulfonylurea (A10BB), DPP-4 inhibitors (A10BH), GLP-1 analogues (A10BJ), SGLT2 inhibitors (A10BK), iron supplementation |
|  | Laboratory values | Hemoglobin, HbA1c, BMI, albumin, CRP, vitamin B12, ferritin |
| Analysis window | Follow-up | 30–365 days after index |
